# Increased small extracellular vesicle levels and decreased miR‐126 levels associated with atrial fibrillation and coexisting diabetes mellitus

**DOI:** 10.1002/clc.24115

**Published:** 2023-08-07

**Authors:** Panjaree Siwaponanan, Pontawee Kaewkumdee, Payalak Sudcharee, Suthipol Udompunturak, Nusara Chomanee, Kamol Udol, Kovit Pattanapanyasat, Rungroj Krittayaphong

**Affiliations:** ^1^ Research Department Siriraj Center of Research Excellence in Microparticles and Exosomes in Disease, Faculty of Medicine Siriraj Hospital, Mahidol University Bangkok Thailand; ^2^ Department of Medicine Division of Cardiology, Faculty of Medicine Siriraj Hospital, Mahidol University Bangkok Thailand; ^3^ Research Department Research Group and Research Network Division, Faculty of Medicine Siriraj Hospital, Mahidol University Bangkok Thailand; ^4^ Department of Pathology Faculty of Medicine Siriraj Hospital, Mahidol University Bangkok Thailand; ^5^ Department of Preventive and Social Medicine Faculty of Medicine Siriraj Hospital, Mahidol University Bangkok Thailand

**Keywords:** atrial fibrillation, diabetes mellitus, microRNAs, small extracellular vesicles

## Abstract

**Background:**

Atrial fibrillation (AF) is the most prevalent cardiac arrhythmia. Diabetes mellitus (DM) is one of the risk factors for the development of stroke and thromboembolism in patients with AF. Early identification may reduce the incidence of complications and mortality in AF patients.

**Hypothesis:**

AF patients with DM have different pattern of small extracellular vesicle (sEV) levels and sEV‐derived microRNA (miRNA) expression compared with those without DM.

**Methods:**

We compared sEV levels and sEV‐miRNA expression in plasma from AF patients with and without DM using nanoparticle tracking analysis and droplet digital polymerase chain reaction, respectively.

**Results:**

We observed a significant increase in total sEV levels (*p* = .004) and a significant decrease in sEV‐miR‐126 level (*p* = .004) in AF patients with DM. Multivariate logistic regression analysis revealed a positive association between total sEV levels and AF with DM (*p* = .019), and a negative association between sEV‐miR‐126 level and AF with DM (*p* = .031). The combination of clinical data, total sEVs, and sEV‐miR‐126 level had an area under the curve of 0.968 (*p* < .0001) for discriminating AF with DM, which was shown to be significantly better than clinical data analysis alone (*p* = .0368).

**Conclusions:**

These results suggest that an increased level of total sEV and a decreased sEV‐miR‐126 level may play a potential role in the pathophysiology and complications of AF with DM, especially endothelial dysfunction, and can be considered as an applied biomarker for distinguishing between AF with and without DM.

## Graphical abstract

This study investigated the association of small extracellular vesicles levels (sEVs) and sEV‐microRNA (miRNA) expression in atrial fibrillation (AF) coexisting with diabetes mullites (DM). We analyzed plasma from age‐ and gender‐matched samples of 2 cohorts (AF with DM and AF without DM). Enriched sEVs were isolated from plasma using PEG precipitation and purified by size exclusion chromatography. sEV levels were measured by nano tracking analysis (NTA) and expression of sEV‐miRNAs was determined by droplet digital PCR. The results showed that increased levels of total sEV and a decreased sEV‐miR‐126 level were associated with the AF coexisting with DM, suggesting that these may play a potential role in the pathophysiology and complications of AF with DM, especially endothelial dysfunction.

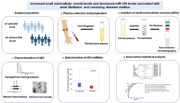

## 1 INTRODUCTION

Nonvalvular atrial fibrillation (AF) is the most common type of sustained cardiac arrhythmia. AF results from abnormal electrical conduction and remodeling of atrial tissues, which influences irregular and fast atrial contractions. The trends in AF prevalence and incidence are rising globally, and they are expected to continue increasing in the future.^1^ Diabetes mellitus (DM) is an independent risk factor for AF and 30% of AF cases occur in diabetes patients.^2^ Coexisting AF and DM increase the risk of cardiovascular mortality, stroke, thromboembolism, chronic kidney disease, and heart failure (HF).^3^, ^4^ The risk of death may be 25−66% higher in AF patients with DM than in nondiabetic ones.^5^

MicroRNA (miRNAs) are single‐stranded noncoding RNAs that are approximately 22 nucleotides in length that play a crucial role in post‐transcriptional gene regulation by inhibiting mRNA translation or by interfering with mRNA stability. miRNAs control several biological processes, including cell proliferation, cell apoptosis, and cell differentiation. Many miRNAs have been implicated in the pathogenesis of diabetes and its related cardiovascular complications. miR‐320 was reported to be an antiangiogenic and proapoptotic miRNA in the cardiovascular system of diabetes patients.^6^ miR‐126‐3p levels are highly expressed in endothelial cells (ECs), and they help to regulate EC functions.^7^ miR‐126‐3p expression was shown to downregulate in intermediate‐age human umbilical ECs (HUVECs) grown in high‐glucose conditions.^8^ Overexpression of miR‐30c‐5p and miR‐181a inhibited cardiomyocyte hypertrophy and apoptosis in high‐glucose‐treated cardiomyocytes.^9^ miR‐146a is a regulator of endothelial inflammation in hyperglycemic conditions via modulation of IRAK‐1.^10^ miRNAs can also be detected in biological fluids, and these miRNAs are called extracellular/circulating miRNAs. The release mechanism of miRNAs has been described in three pathways, including extracellular vesicles (EVs) (apoptotic bodies, exosomes, and microvesicles), high‐density lipoprotein, and protein complex (AGO2).^11^

EVs are nanoparticle lipid bilayers that are released from most cells into the extracellular space. EVs act as vehicles that transport bioactive molecules, such as lipids, proteins, metabolites, and nucleic acids, in cell‐to‐cell communication.^12^ The International Society for EVs has proposed classifying EVs based on size, as follows: small EVs (sEV) (<100 nm or <200 nm) and medium/large EVs (>200 nm).^13^ miRNA sorting into EVs is a selective packaging mechanism that can reflect the cellular response to pathophysiological conditions. Therefore, EV‐derived miRNAs (EV‐miRNAs) are emerging as diagnostic and prognostic biomarkers for many diseases.^14^, ^15^, ^16^

In this study, we set forth to investigate the difference in sEV levels and sEV‐derived miRNA expression between AF patients with and without DM. We hypothesized that our findings would reveal the basic knowledge to apply sEV and sEV‐miRNAs to be potential biomarkers for AF with DM.

## 2 MATERIAL AND METHODS

### 2.1 Study population and subject enrollment

**Figure 1:**
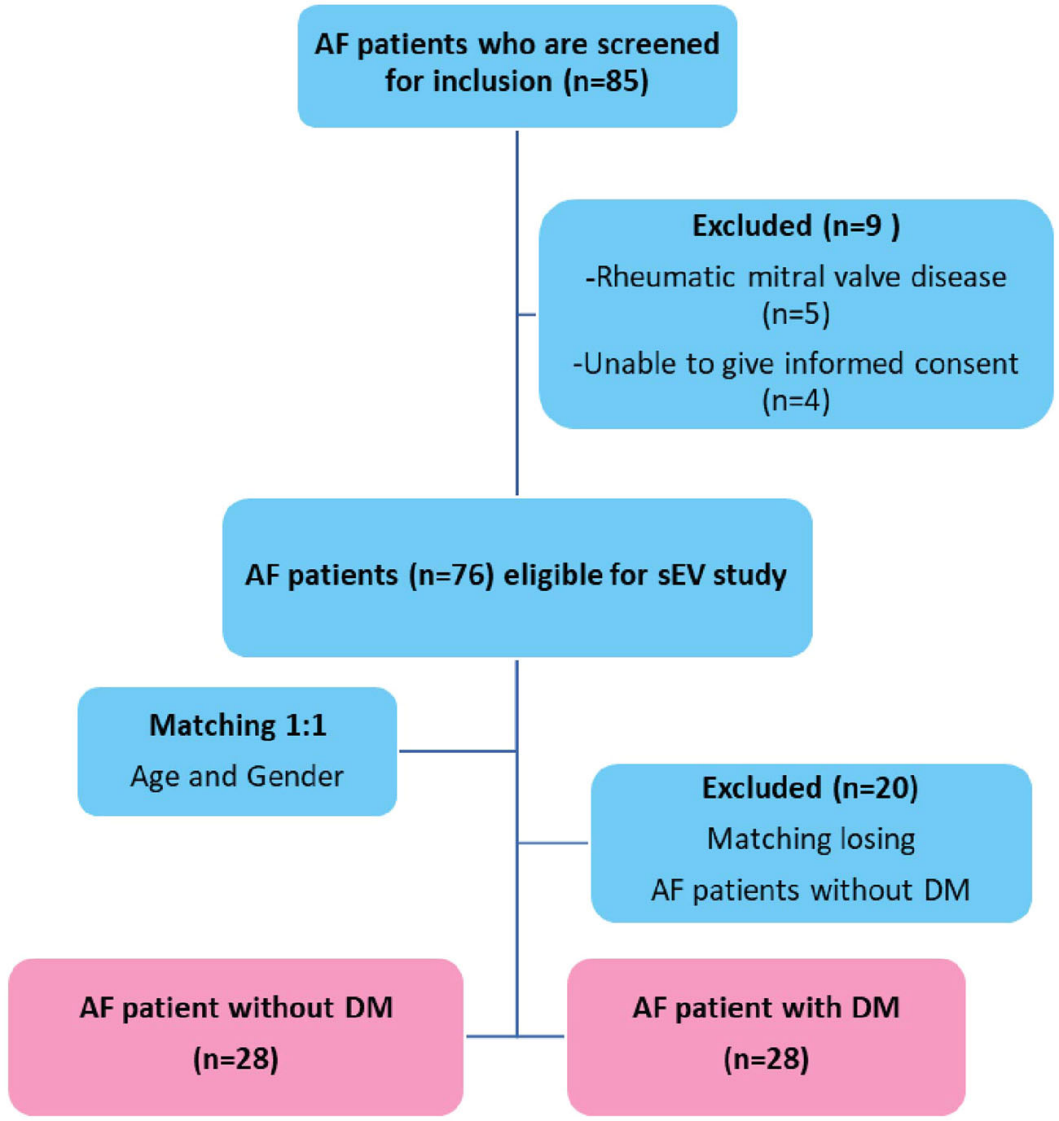
A flowchart of the enrollment of the study cohort.

Nonvalvular AF patients were prospectively recruited from the Division of Cardiology of the Department of Medicine and from the Department of Preventive and Social Medicine, Faculty of Medicine Siriraj Hospital, Mahidol University, Bangkok, Thailand during the November 2019 to February 2022 study period. The protocol for this study was approved by the Siriraj Institutional Review Board (SIRB) (COA no. Si 489/2019). Eighty‐five AF patients were enrolled and then nine AF patients were excluded following the exclusion criteria. The exclusion criteria were refusal to provide written informed consent to participate, history of myeloproliferative disorders, thrombocytopenia, ischemic stroke within the previous 3 months, malignancy, rheumatic mitral valve disease, acute infectious or inflammatory disease, and pregnancy. The sample size was calculated based on information from the previous study using nQuery software.^17^ Age and gender were matched between the AF with DM and AF without DM. The AF with DM group comprised 28 AF patients who had been diagnosed with DM according to the World Health Organization (WHO) diagnostic criteria (2 new‐onset AF, 17 paroxysmal AF, 6 persistent AF, and 3 permanent AF). The AF without DM group comprised 28 AF patients without baseline DM (2 new‐onset AF, 19 paroxysmal AF, 2 persistent AF, and 5 permanent AF). A flowchart of the enrollment of the study cohort was shown in Figure 1.

### 2.2 Blood processing and sEV isolation

EDTA‐anticoagulated peripheral blood was centrifuged at 1500 times gravity (*g*) for 15 minutes to isolate platelet‐poor plasma (PPP) followed by storage at −80°C until analysis. sEV isolation was modified from a previously described method.^18^ Briefly, PPP was centrifuged at 17 000*g* for 2 minutes at 4°C and the large EVs were then removed via centrifugation at 17 000*g* for 45 minutes at 4°C. The large EV‐free plasma was diluted with an equal volume of phosphate‐buffered saline (PBS) and the mixture was filtered through 0.22 μm syringe filters (Corning Life Sciences). The diluted plasma was mixed with 20% weight/volume (w/v) polyethylene glycol 6000 (PEG6000) (Sigma‐Aldrich Corporation) in diluent buffer (200 mM NaCl, 10 mM EDTA, 200 mM Tris‐HCl, pH 7.0) and rotated end‐over‐end at 4°C for 24 h. The samples were then centrifuged at 4000*g* for 30 minutes and the pellets were resuspended in 500 µL PBS. sEV‐enriched samples were purified using size exclusion chromatography (SEC) columns (qEV original with 70 nm pore size; Izon Science, Christchurch, New Zealand) following the manufacturer's instructions. sEV‐enriched samples were applied on top of qEV columns and 0.5 mL fractions were collected. EV‐rich fractions (7−12) were pooled and concentrated using an Amicon Ultra‐4 10 kDa centrifugal filter device (Merck Millipore). sEVs were characterized using Nanoparticle tracking analysis (NTA), Transmission electron microscopy (TEM), and Western blot (WB) analysis (Supporting Information Materials).

### 2.3 miRNA selection

Our study selected miRNAs from the literature research, which involve the crucial pathophysiology of cardiovascular diseases coexisting with DM and hyperglycemic condition, including cell apoptosis, angiogenesis, diabetic cardiomyopathy, oxidative stress, fibrosis, and inflammations. miR‐320 is upregulated in the heart in hyperglycemic conditions. miR‐320a‐3p has been reported to regulate cardiac dysfunction in DM by activating the transcription of fatty acid metabolic genes to cause lipotoxicity in the heart.^19^ miR‐126‐3p plays a crucial role in the maintenance of vascular integrity, which promotes vascular cell survival. Hyperglycemia‐induced miR‐126 underexpression influences the angiogenic pathway by regulating SPRED1 and PIK3R2 expression.^20^ This mechanism induces endothelial dysfunction and promotes the development of diabetic complications. miR‐30c‐5p improves glucose utilization and reduces the excessive reactive oxygen species (ROS) production and subsequently attenuates cardiomyocyte apoptosis and cardiac dysfunction in diabetic cardiomyopathy.^21^ miR‐146a was associated with elevated inflammatory factor and extracellular matrix protein production and cardiac functional alterations in the diabetic heart.^22^

### 2.4 Droplet digital polymerase chain reaction (ddPCR)

EV‐miRNA expression was quantified usig a ddPCR™ system (Bio‐Rad Laboratories). sEV‐RNA was extracted using TRIzol LS® Reagent (Life Technologies) following the manufacturer's protocol. sEV‐RNA concentration was measured using a NanoDrop™ 8000 Spectrophotometer (Thermo Fisher Scientific). Five microliter of total RNA was reverse‐transcribed using a miRCURY LNA™ Universal RT miRNAs PCR, according to the manufacturer's instructions. Detailed information about the primers and thermal cycling conditions used for the evaluated miRNAs is presented in Supporting Information: Table 1. The cartridges were placed inside a QX200 Droplet Generator (Bio‐Rad Laboratories), and then the droplet mixtures were transferred to a ddPCR™ 96‐well PCR plate (Bio‐Rad Laboratories). PCR amplification was performed using a C1000 Touch Thermal Cycler (Bio‐Rad Laboratories). A no template control was included in every assay. The PCR‐positive and negative droplets were read using a QX200 Droplet Reader (Bio‐Rad Laboratories). QuantaSoft software (Bio‐Rad Laboratories) was used to calculate the concentration of miRNAs, and the results are presented as the number of copies per microliter of PCR reaction. Synthetic miRNA (Cel‐miR‐39‐3p) was added to all samples to check RNA extraction efficiency.

### 2.5 Statistical analysis

The data were analyzed using PASW Statistics version 18 (SPSS, Inc.) and MedCalc® Statistical Software version 20 (MedCalc Software Ltd.). Normally distributed continuous variables were analyzed using unpaired *t*‐test, and the results are presented as the mean plus/minus standard deviation (SD). Non‐normally distributed continuous data were compared using Mann−Whitney *U* test. Categorical variables were compared using *χ* ^2^ test. Univariate logistic regression analysis was performed to evaluate for significant association between clinical parameters, total sEV levels, sEV smaller than 100 nm and sEV‐miR‐126 levels and AF with DM. Multivariate logistic regression analysis was then performed to identify independent predictors of AF with DM. Total sEV, sEV smaller than 100 nm, and sEV‐miR‐126 were then evaluated for association with AF with DM by adjusting for possible confounding variables. The independent factors identified from multivariate analysis were then used to generate four additional prediction models (for a total of 5 models) using logistic regression analysis. Models 2−5 included model 1 and one or more of the identified independent variables. The data from each of the 5 models specific to each model's ability to predict AF with DM was then used to generate receiver operating characteristic (ROC) curves. A significant difference was defined as a *p*‐value less than .05.

## 3 RESULTS

### 3.1 Patient characteristics

The baseline demographic and clinical characteristics compared between the AF with DM group (*n* = 28) and the AF without DM group (*n* = 28) are shown in Table 1. There were significantly less patients taking warfarin in the AF with DM group than in the AF without DM group (64.3% vs. 92.9%, respectively; *p* = .009). AF patients with DM had a significantly higher mean fasting blood glucose than AF patients without DM (127.43 ± 35.22 vs. 97.84 ± 10.58 mg/dL, respectively; *p* < .001). HDL‐C and calculated LDL‐C were both significantly lower in AF patients without DM than in patents with DM (HDL‐C: 49.62 ± 13.17 vs. 58.76 ± 13 mg/dL, *p* = .012; and, calculated LDL‐C: 74.15 ± 20.27 vs. 88.97 ± 26.67 mg/dL, *p* = .023—both respectively). All other variables were nonsignificantly different between groups.

**Table 1:** Baseline demographic and clinical characteristics compared between the AF with DM group and the AF without DM group.

Characteristics	AF with DM (*n* = 28)	AF without DM (*n* = 28)	*p* Value
Age (years)	71.21 ± 8.49	71.82 ± 7.62	.779
Male gender	17 (47.2%)	19 (52.8%)	.577
Body mass index (kg/m^2^)	24.93 ± 4.47	26.00 ± 3.03	.299
Systolic blood pressure (mmHg)	133.86 ± 22.75	136.93 ± 18.85	.584
Diastolic blood pressure (mmHg)	73.43 ± 13.09	77.64 ± 14.08	.251
Heart rate (bpm)	75.32 ± 16.24	71.57 ± 14.48	.366
Medical history			
‐ Hypertension	25 (89.3%)	24 (85.7%)	.686
‐ Dyslipidemia	20 (71.4%)	16 (57.1%)	.265
‐ Congestive heart failure	12 (42.9%)	8 (28.6%)	.265
‐ Stroke	3 (10.7%)	4 (14.3%)	.686
‐ History of bleeding	5 (17.9%)	6 (21.4%)	.737
Smoking status			
‐ Ex‐smoker	13 (46.4%)	14 (50.0%)	.964
‐ Current smoker	1 (3.6%)	1 (3.6%)	
‐ Never smoked	13 (46.4%)	14 (50.0%)	
Medications			
‐ Beta‐blocker	21 (75.0%)	20 (71.4%)	.763
‐ Calcium channel blocker	10 (35.7%)	11 (39.3%)	.783
‐ ACE inhibitors	5 (17.9%)	7 (25.0%)	.515
‐ ARB	7 (25.0%)	10 (35.7%)	.383
‐ Statin	13 (46.4%)	17 (60.7%)	.284
‐ Warfarin	18 (64.3%)	26 (92.9%)	***.009***
‐ Aspirin	3 (10.7%)	2 (7.1%)	.639
Laboratory parameters			
‐ Fasting blood sugar (mg/dL)	127.43 ± 35.22	97.84 ± 10.58	***<.001***
‐ Total cholesterol (mg/dL)	153.08 ± 30.50	168.52 ± 33.11	.075
‐ Triglyceride (mg/dL)	111.00 (83.50−154.75)	93.00 (68.50−114.50)	.084
‐ HDL‐C (mg/dL)	49.62 ± 13.17	58.76 ± 13.31	***.012***
‐ Calculated LDL‐C (mg/dL)	74.15 ± 20.27	88.97 ± 26.67	***.023***
‐ Serum creatinine (mg/dL)	1.20 (0.97−1.88)	1.06 (0.97−1.20)	.166
‐ INR level	1.98 (1.48−2.37)	2.19 (1.78−2.51)	.207

*Note*: Data presented as number and percentage, mean plus/minus standard deviation, or median and interquartile range. A *p* < .05 indicates statistical significance.

Abbreviations: ACE, angiotensin‐converting enzyme; AF, atrial fibrillation; ARB, angiotensin receptor blockers; bpm, beats per minute; DM, diabetes mellitus; HDL‐C, high‐density lipoprotein cholesterol; INR, international normalized ratio; LDL‐C, low‐density lipoprotein cholesterol.

### 3.2 Characterization and concentration of sEVs

**Figure 2:**
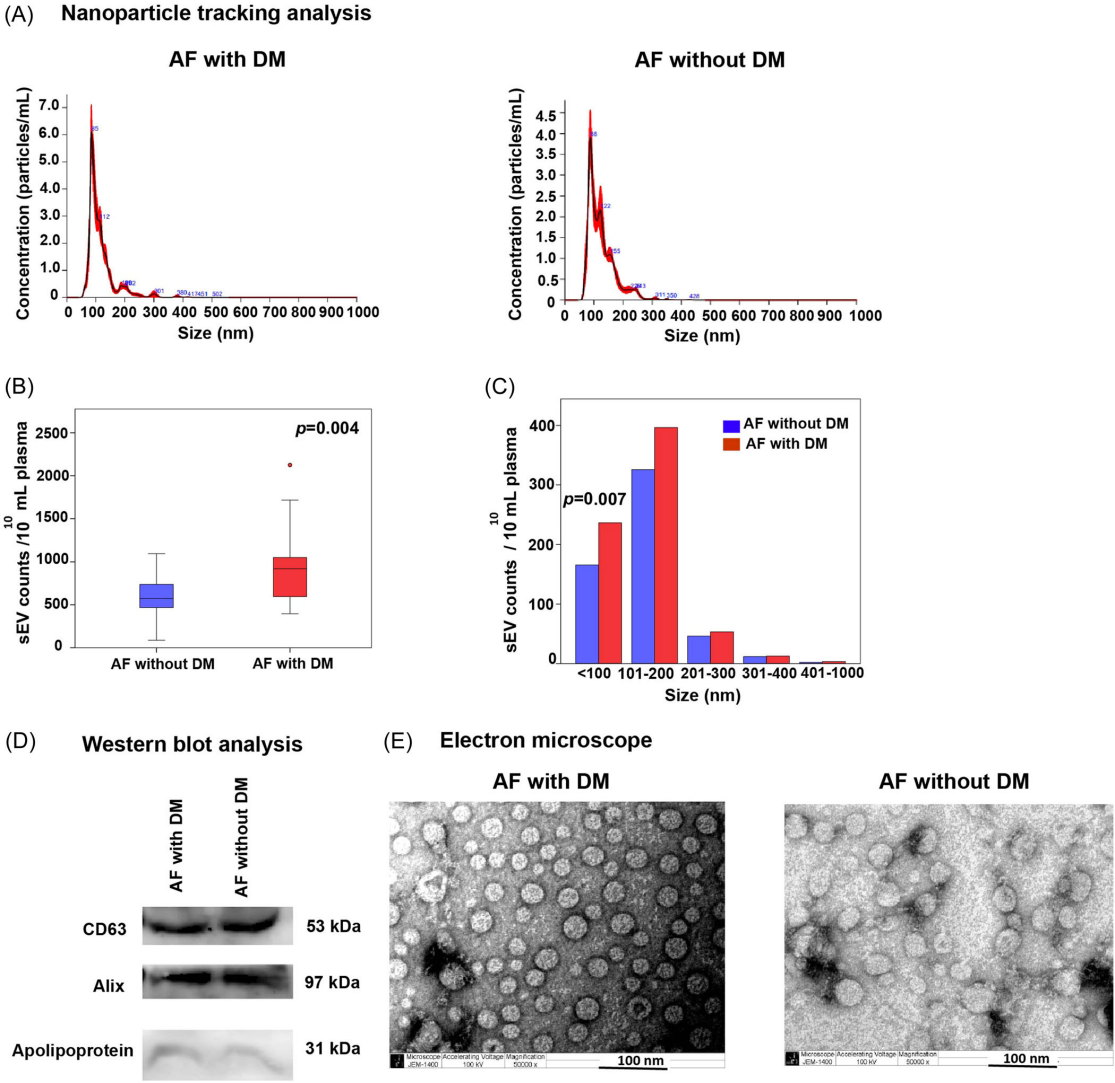
Characterization of small extracellular vesicles (sEVs). Plasma sEVs from atrial fibrillation (AF) patients with diabetes mellitus (DM) and without DM were characterized via nanoparticle tracking analysis (NTA), Western blot analysis, and transmission electron microscopy (TEM). (A) NTA showing the size and concentration of sEVs in the AF with and without DM groups. (B) NTA showing the total sEV count compared between AF patients with DM (n = 28) and without DM (*n* = 28). (C) NTA showing the total sEV count compared between groups and stratified by sEV size subcategory. (D) Western blot analysis showing the sEV markers CD63 and Alix and the contaminant lipoprotein marker apolipoprotein in sEV samples (30 µg protein per lane). (E) The morphologies of sEVs from both groups were visualized by TEM (scale bar: 100 nm, ×50 000 magnification). A *p* < .05 indicates statistical significance.

sEVs were isolated from patient plasma and characterized using NTA, TEM, and WB analysis. The size of isolated sEVs ranged from 50 to 400 nm in diameter, but the majority were 100 nm in size (Figure 2A). The median concentration of sEVs from AF patients with DM was 919.79 × 10^10^ particles/mL [IQR: 577.08−1050.00], which was significantly higher than that from AF patients without DM (571.88 × 10^10^ particles/mL [IQR: 462.50−740.63]; *p* = .004) (Figure 2B). sEVs were categorized according to size and analyzed. The results showed sEVs sized smaller than 100 nm to be significantly elevated in AF patients with DM compared to AF patients without DM (236.22 × 10^10^ particles/mL [IQR: 161.25−476.28] vs. 165.75 × 10^10^ particles/mL [IQR: 113.72−248.22], respectively; *p* = .007) (Figure 2C). There was no significant difference in any of the other sizes of sEVs between groups. WB analysis revealed the expression of the EV markers Alix and CD63, as well as of the coisolate contamination marker Apolipoprotein in sEV samples from both groups (Figure 2D). Investigation of sEV morphology using TEM revealed that plasma sEVs from both groups had cup‐shaped vesicles, which is the typical morphology of sEVs (Figure 2E).

### 3.3 Expression of sEVs‐derived‐miRNAs (sEV‐miRNAs)

**Figure 3:**
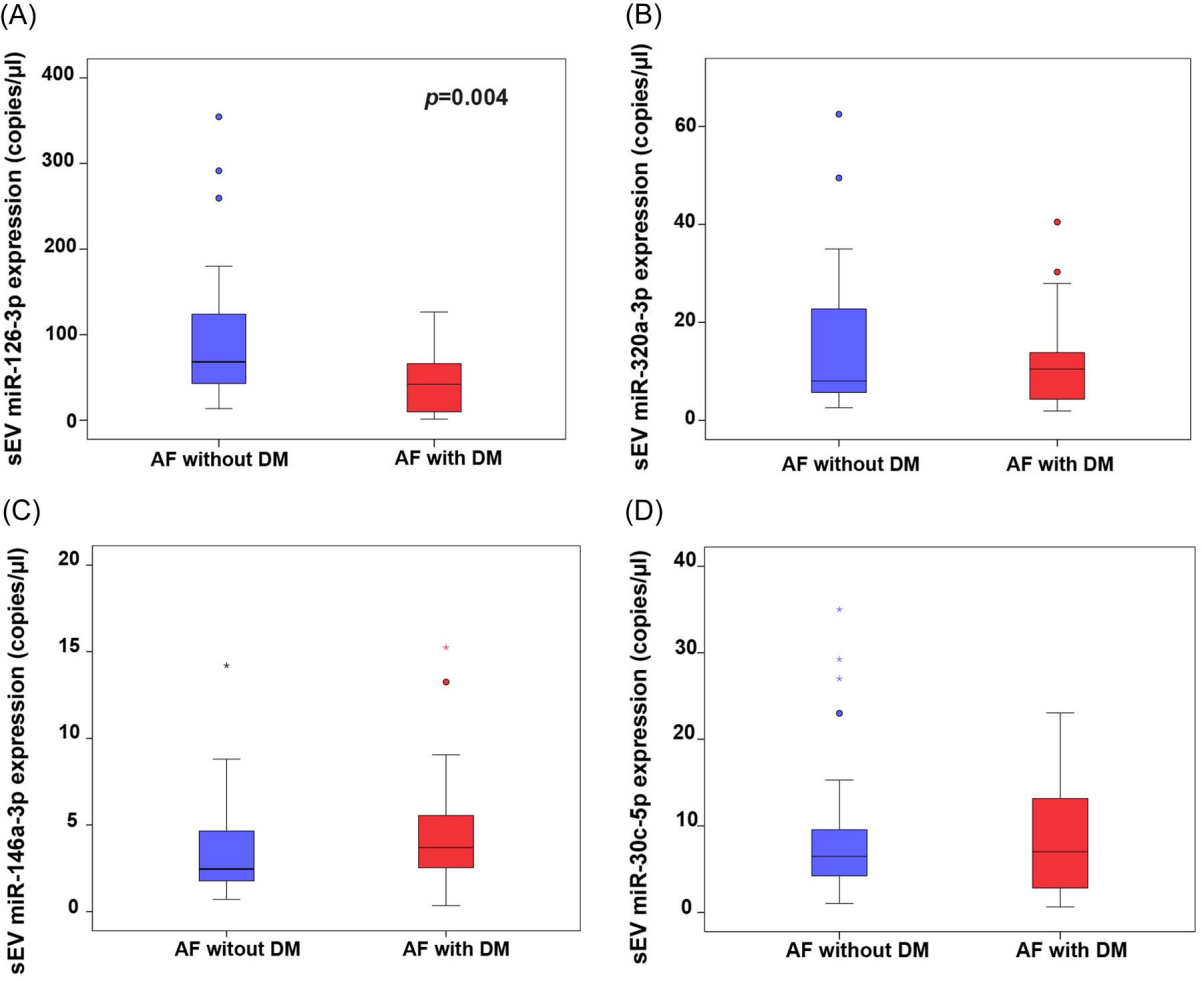
Absolute quantification by droplet digital polymerase chain reaction (ddPCR) of microribonucleic acid (miRNA) expression in small extracellular vesicles (sEVs). (A) miR‐126‐3p, (B) miR‐320a‐3p, (C) miR‐146a‐3p, and (D) miR‐30c‐5p in sEVs from and compared between atrial fibrillation (AF) patients with diabetes mellitus (DM) and without DM. Differences between groups were analyzed using Mann−Whitney *U* test for independent samples. Data shown as median and interquartile range, and a *p* < .05 indicates statistical significance.

To study the difference in the expression of sEV‐miRNAs between AF patients with and without DM, we measured the expression of these miRNAs using ddPCR, and the results showed the levels of miR‐126‐3p expression to be significantly decreased in AF patients with DM compared to AF patients without DM (42.00 copies/µL [IQR: 8.85−67.13] vs. 68.25 copies/µL [IQR: 42.00−125.25], respectively; *p* = .004). The expression levels of the other miRNAs were not significantly different between groups, as shown in Figure 3.

### 3.4 Levels of total sEVs and sEV‐miR‐126‐3p expression associated with AF with DM

**Figure 4:**
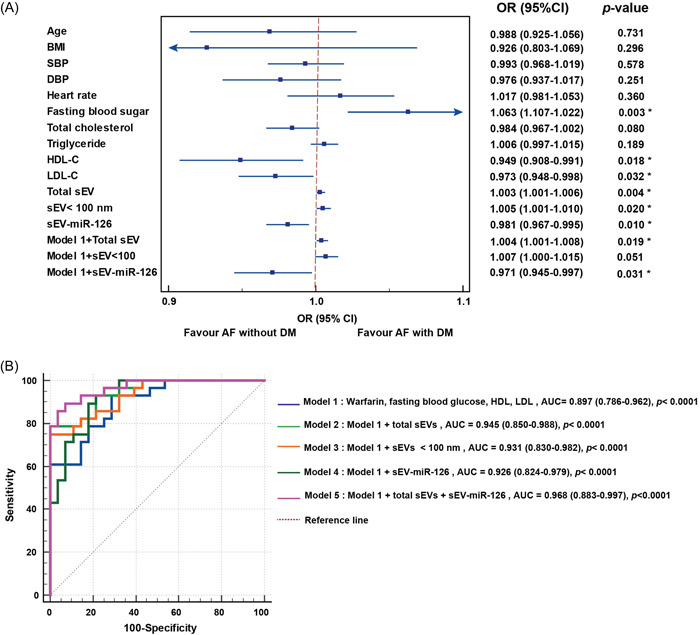
Analysis for predictors of atrial fibrillation (AF) with diabetes mellitus (DM) or AF without DM, and a comparison among models for predicting AF with DM. (A) Univariate logistic regression analysis was performed to identify significant predictors/factors that favor either AF with DM or AF without DM. (B) To explore the discriminatory ability of total sEVs and sEV‐miR‐126‐3p levels to predict AF with DM, we analyzed five multivariate logistic regression models. The results of those analyses were used to generate receiver operating characteristic (ROC) curves and the area under the ROC curve (AUC) was calculated to determine the predictive sensitivity and specificity of each model. The AUC for all 5 models was 0.897 or higher, and the *p*‐value for all 5 models was *p* < .0001; however, model 5, which included clinical data, total sEVs, and sEV‐miR‐126 levels, had the highest AUC (0.968). Moreover, model 5 or the combination of model 1 with sEV and sEV‐miR‐126 showed a significant increase in AUC compared to model 1 alone (*p* = .038). A *p*‐value < .05 indicates statistical significance. (BMI, body mass index; CI, confidence interval; DBP, diastolic blood pressure; HDL‐C, high‐density lipoprotein cholesterol; LDL‐C, low‐density lipoprotein cholesterol; OR, odds ratio; SBP, systolic blood pressure; sEV, small extracellular vesicle).

Univariate logistic regression analysis was employed to evaluate for associations between possible risk factors and AF with DM. That analysis revealed significant positive associations between AF with DM as the outcome and fasting blood sugar levels (odds ratio [OR]: 1.063, 95% confidence interval [CI]: 1.107−1.022; *p* = .003), total sEV levels (OR: 1.003, 95% CI: 1.001−1.006; *p* = .004), and sEV smaller than 100 nm (OR: 1.005, 95% CI: 1.001−1.010; *p* = .020). AF with DM was shown to be significantly negatively associated with sEV‐miR‐126‐3p levels (OR: 0.981, 95% CI: 0.967−0.995; *p* = .010), HDL‐C levels (OR: 0.949, 95% CI: 0.909−0.991; *p* = .018), and calculated LDL‐C levels (OR: 0.973, 95% CI: 0.948−0.998; *p* = .032), as shown in the forest plot in Figure 4A. Multivariate logistic regression analysis was then performed to confirm the relationship between AF with DM and total sEVs, sEV smaller than 100 nm, and sEV‐miR‐126‐3p levels. The results of that analysis showed total sEVs and sEV‐miR‐126‐3p levels to be independently associated with AF with DM after adjusting for possible confounding factors (model 1: fasting blood sugar, HDL‐C, calculated LDL‐C levels, and taking warfarin) (Figure 4A). To further explore the ability of total sEVs and sEV‐miR‐126‐3p levels to distinguish AF with DM, we analyzed five logistic regression models for predicting AF with DM, as follows: Model 1: fasting blood sugar, HDL‐C, calculated LDL‐C levels, and taking warfarin; Model 2: model 1 + total sEV; Model 3: model 1 + sEV smaller than 100 nm; Model 4: model 1 + miR‐126‐3p; and, Model 5: model 1 + total sEV levels + miR‐126‐3p. The results of those analyses were used to generate ROC curves and the area under the ROC curve (AUC) was calculated to determine the predictive sensitivity and specificity of each model (Figure 4B). The AUC values for each of the 5 models are shown, as follows: AUC: 0.897 (95% CI: 0.786−0.962), *p* < .0001 for model 1; AUC: 0.945 (95% CI: 0.850−0.988), *p* < .0001 for model 2; AUC: 0.931 (95% CI: 0.830−0.982), *p* < .0001 for model 3; AUC: 0.926 (95%CI: 0.824‐0.979), *p* < .0001 for model 4; and AUC: 0.968 (95% CI: 0.883−0.997), *p* < .0001 for model 5. Model 5 or the combination of model 1 with sEV and sEV‐miR‐126 had the highest AUC, and showed a significant increase in AUC compared to model 1 alone (*p* = .038). The sensitivity and specificity were 92.86% and 71.43% for model 1; 78.57% and 100% for model 2; 75.0% and 100% for model 3; 96.43% and 75.0% for model 4; and, 89.29% and 92.86% for model 5.

## 4 DISCUSSION

DM is a common metabolic disorder that predisposes AF patients to cardiovascular events and mortality.^23^ AF patients with DM have a worse prognosis, lower quality of life, and increased risk of death and hospitalizations compared to AF patients without DM.^24^ The relationship between sEV levels and/or sEV‐miRNA expression and AF with DM is not well understood. This is the first study to investigate the difference in sEV levels and sEV‐derived miRNA expression between AF with and without DM. We found high sEV levels in AF patients with DM, especially sEVs sized smaller than 100 nm. This finding is consistent with a previous study that found a significantly increased sEV concentration in diabetes patients compared to euglycemic individuals.^25^ The mechanisms underlying DM‐related AF, such as oxidative stress, insulin resistance, and inflammation, may support sEV generation.^26^ ROS activate proapoptotic p53, which promotes increased shedding of EVs.^27^ Elevated inflammatory markers, such as C‐reactive protein, tumor necrosis factor alpha (TNF‐α), and interleukin‐6, were observed in diabetic patients.^28^ TNF‐α treatment significantly promoted the release of EVs from mouse astrocytes and HUVECs.^29^ Several insulin signaling proteins were identified in the EVs of diabetic patients, which suggests that insulin resistance increases EV secretion.^25^

On the other hand, high levels of sEVs can promote the pathophysiology of diabetes and diabetic complications. High levels of cluster of differentiation 40 (CD40) and its corresponding ligand (CD40L), which influence EC activation and monocyte recruitment leading to the acceleration of atherosclerosis development, have been observed in diabetic patients.^30^ An increased abundance of antiangiogenic factors was found in EVs from diabetic patients. RANTES is a chemokine that is strongly related to retinopathy and angiopathy, and TIMP1 and TIMP2 play roles in the inhibition of EC morphogenesis.^31^

In addition to DM, the pathophysiology of AF contributes to sEV release and sEVs can promote AF development. Prolonged rapid atrial pacing in canine model increased the release of plasma sEVs and induced atrial fibrosis.^32^ Our research group previously reported increased levels of miR‐106b‐3p, miR‐590‐5p, miR‐339‐3p, miR‐378‐3p, miR‐328‐3p, and miR‐532‐3p in large EVs, suggested that these miRNAs may play a role in arrhythmogenesis and/or structural remodeling in AF.^33^ Epicardial fat (eFat)‐derived EVs from AF patients were shown to influence inflammation, atrial fibrosis, and arrhythmogenesis.^34^

**Figure 5:**
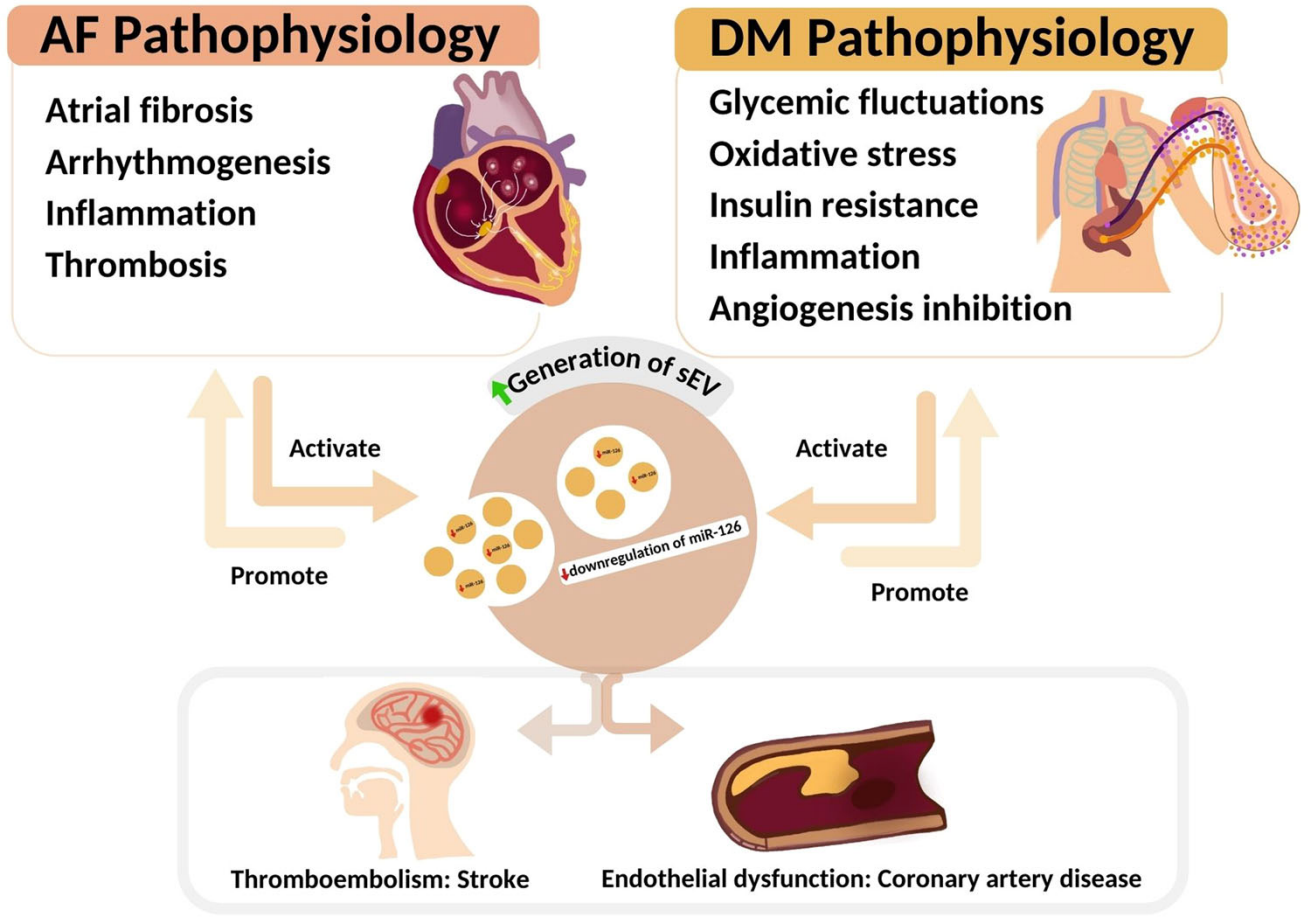
The putative mechanisms involved in the relationship between small extracellular vesicle (sEV) generation, sEV‐miR‐126‐3p expression, and atrial fibrillation (AF) with diabetes mellitus (DM). The pathophysiologies of AF and DM stimulate sEV generation and downregulation of miR‐126‐3p, which influences the progression of both AF and DM, as well as cardiovascular complications, including thromboembolism leading to stroke and endothelial dysfunction leading to coronary artery disease.

The role of EVs in thrombogenicity has been described in AF and is associated with a risk of thromboembolic complications, including ischemic stroke and systemic thromboembolism. EV subtypes (phosphatidyl serine positive EVs, erythrocyte‐derived EVs, platelet‐derived EVs, and leukocyte‐derived EVs) were found to be significantly higher in patients with AF along with an increased risk of stroke as compared to matched controls.^35^ Tissue factor (TF)‐bearing EVs were found to be significantly higher in the plasma of AF patients compared to controls. This suggests that TF‐bearing EVs may influence increased thrombogenicity in AF patients.^36^ Taken together, these data strongly suggest that high levels of EVs exacerbate the pathophysiology and complications of AF with DM, including endothelial dysfunction and thromboembolism (Figure 5).

miR‐126, also called angiomiR‐126, is highly expressed in ECs and platelets, and acts as an important regulator for the maintenance of endothelial homeostasis and vascular integrity. Circulating miR‐126 acts as a potential biomarker for vascular damage and cardiac injury in cardiovascular diseases, including myocardial infarction (AMI),^37^ heart failure,^38^ coronary artery disease (CAD),^39^ and AF.^40^ Patients with diabetes were reported to have decreased levels of circulating miR‐126, which was found to be associated with endothelial dysfunction.^41^ In AF, serum miR‐126 levels were significantly lower in patients with permanent or persistent AF than in those with paroxysmal AF, and they were negatively associated with LVEF, the logarithm of NT‐proBNP, left atrial diameter, and cardiothoracic ratio.^40^ Overall, decreased circulating miR‐126 levels may underlie changes in vascular and heart function in AF patients with DM.^40^, ^41^

Endothelial dysfunction is an important factor in the development of vascular complications in patients with DM. The mechanisms underlying this phenomenon involve hyperglycemia, insulin resistance, and oxidative stress.^42^ Endothelial‐specific miR‐126 is the most highly expressed miRNA in endothelial‐derived microparticles, and it promotes the migration and proliferation of ECs. Low miR‐126 levels have been found in apoptotic bodies and microvesicles in DM patients.^41^ High glucose‐treated endothelial‐derived EVs contained a low amount of miR‐126, which resulted in the loss of the regenerative capacity of ECs.^43^ Our study found decreased sEV‐miR‐126 levels in AF patients with DM. This may reflect the underlying mechanisms involved in sEV‐miR‐126 regulation and endothelial dysfunction in AF patients with DM. Hyperglycemia may result in endothelial dysfunction and loss of miR‐126 expression in EVs, which contributes to impaired angiogenesis and/or endothelial regeneration in AF patients with DM.^41^, ^44^

There were no differences in miR‐320a‐3p, miR‐146a‐5p, or miR‐30c‐5p levels in sEVs between AF patients with DM and without DM, which may be due to the effect of drug treatment. Over half (53%) of AF patients with DM in our cohort were treated with anti‐diabetes drugs, which were shown to influence the generation, release, and composition of EVs in type 2 DM patients. Furthermore, anti‐diabetes drugs were found to restore the levels of many miRNAs in EVs close to those observed in healthy controls.^45^

### 4.1 Limitations

This study has some mentionable limitations. First, although our sample size per group satisfied the minimum required by our sample size calculation, further evaluation of our findings needs to be conducted in a much larger and more racially diverse patient population to verify that total sEV level and sEV‐miR‐126 levels accurate predict AF with DM. Second, we only investigated miRNAs that were reported to have known functions related to the pathophysiology of DM and cardiovascular diseases. An investigation to identify new miRNAs should be conducted using small RNA sequencing. Third, all 56 of the AF patients enrolled in this study were Asian (Thai), which could limit the generalizable of our findings to AF patients of other races. Fourth, the role(s) of miR‐126‐3p in the development and complications of AF and DM need to be explored in future studies. Last, the prospective cohort study (such as the incidence of AF in patients with DM or vice versa) needed to be investigated in the future to understand the pattern of sEV levels and sEV‐miRNA expression in parallel with the development of coexisting DM with AF.

## 5 CONCLUSION

The results of this study strongly suggest that the levels of total sEV and sEV‐miR‐126 associated with AF and coexisting DM. We observed a significant increase in total sEV levels and a significant decrease in sEV‐miR‐126 levels in AF patients with DM. This alteration may be the one of mediators that accelerates the development of pathophysiology and complications in AF and coexisting with DM, especially endothelial dysfunction.

## AUTHOR CONTRIBUTIONS

*Conceptualization*: Panjaree Siwaponanan and Rungroj Krittayaphong. *Methodology*: Panjaree Siwaponanan and Nusara Chomanee. *Formal analysis*: Panjaree Siwaponanan and Suthipol Udompunturak. *Investigation*: Panjaree Siwaponanan and Nusara Chomanee. *Validation*: Panjaree Siwaponanan Resources, Panjaree Siwaponanan, Pontawee Kaewkumdee, Kamol Udol and Rungroj Krittayaphong. *Data Curation*: Panjaree Siwaponanan and Pontawee Kaewkumdee. *Writing*—*Original Draft*: Panjaree Siwaponanan; *Writing*—*review & editing*: Panjaree Siwaponanan, Kovit Pattanapanyasat and Rungroj Krittayaphong. *Visualization*: Panjaree Siwaponanan Supervision, Rungroj Krittayaphong. *Project administration*: Panjaree Siwaponanan; *Funding acquisition*: Panjaree Siwaponanan.

## CONFLICT OF INTEREST STATEMENT

The authors declare no conflict of interest.

## Supporting information


**Supplementary Table 1.** miRCURY LNA™ PCR primers and the thermal cycling conditions used for ddPCR.

## Data Availability

The data presented in this study are available on request from the corresponding author. The data are not publicly available due to privacy restrictions.
