# Supplementary material for: Increased small extracellular vesicle levels and decreased miR‐126 levels associated with atrial fibrillation and coexisting diabetes mellitus
Source: Clin Cardiol. 2023 Aug 7;46(11):1326–36. doi: 10.1002/clc.24115 (PMC10642338; doi:10.1002/clc.24115)
Supplement: Supplementary file 1 — Supplementary Table 1. miRCURY LNA™ PCR primers and the thermal cycling conditions used for ddPCR. [file CLC-46-1326-s001.docx]

**Supplementary material**

1. Nanoparticle tracking analysis (NTA)

sEV concentration and size were measured using a NanoSight NS300 system (Malvern Panalytical, Malvern, UK) equipped with a 488 nanometer (nm) laser and NTA software version 3.4 (Malvern Panalytical). sEV samples were diluted (1:200-1:500) in 1 ml of filtered PBS. Samples were analyzed under constant flow conditions (flow rate: 30) at 25°C, and were photographically captured at a camera level of 13-14 and a detection threshold of 5. Five independent measurements (60s each) were obtained for each sample.

2. Transmission electron microscopy (TEM)

Isolated sEVs were fixed in 2% glutaraldehyde (Sigma-Aldrich Corporation) in PBS for 30 minutes at 4°C, and then absorbed onto 200 mesh copper grids with carbon-coated formvar film for 15 minutes (Electron microscope science, Hatfield, PA). The grids were washed with filtered PBS and negatively stained with 2% uranyl acetate (w/v) for 3 minutes. The grids were imaged under a transmission electron microscope (JEM-1400; JEOL Ltd, Tokyo, Japan) at 100 kV.

3. Western blot analysis

The sEV protein was quantified using a bicinchoninic acid assay kit (Pierce; Thermo Fisher Scientific, Waltham, MA, USA), according to the manufacturer's instructions. Thirty μg of total protein was mixed with reducing sample buffer and loaded onto 10% sodium dodecyl sulfate-polyacrylamide gel electrophoresis (SDS-PAGE). Proteins were then transferred to a polyvinylidene difluoride (PVDF) membrane (GE Healthcare Life Sciences, Chicago, IL, USA). The membrane was blocked with 5% w/v non-fat milk in Tris buffered saline plus Tween® 20  (TBST) buffer followed by incubation with the primary antibodies anti-CD63, anti-Alix, and anti-Apolipoprotein A (Abcam, Cambridge, UK) overnight at 4°C. After washing, the membranes were incubated with horseradish peroxidase (HRP)-linked goat anti-rabbit immunoglobulin G (IgG) (Abcam) for 1 hour at room RT. Chemiluminescence detection was performed using Clarity Western ECL Substrate (Bio-Rad Laboratories, Inc, Hercules, CA, USA). The protein bands were visualized using an ImageQuant LAS 4000 system (GE Healthcare Life Sciences).

**Supplementary Table 1.** miRCURY LNA™ PCR primers used for ddPCR

| miRNA ID | miRBase database accession number | Target sequence | miRCURY LNA™ miRNA PCR assay catalog number |
| --- | --- | --- | --- |
| hsa-miR-126-3p | MIMAT0000445 | 5'UCGUACCGUGAGUAAUAAUGCG'3 | YP00204227 |
| hsa-miR-146a-3p | MIMAT0004608 | 5'CCUCUGAAAUUCAGUUCUUCAG'3 | YP00204401 |
| hsa-miR-320a-3p | MIMAT0000510 | 5'AAAAGCUGGGUUGAGAGGGCGA'3 | YP00206042 |
| hsa-miR-30c-5p | MIMAT0000244 | 5'UGUAAACAUCCUACACUCUCAGC'3 | YP00204783 |

**Abbreviations:** ddPCR, droplet digital polymerase chain reaction; LNA, locked nucleic acid**;** miRNA, microribonucleic acid(s); PCR, polymerase chain reaction

The thermal cycling conditions were, as follows: 95°C for 5 minutes, 40 cycles of 95°C for 30 seconds, and 54°C for 1 minute (for miR-146a-3p and miR-30c-5p) or 56°C for 1 minute (for miR-126-3p and miR-320a-3p) (ramping rate reduced to 2%), and three final steps at 4°C for 5 minutes, 90°C for 5 minutes, and 4°C indefinite holds.
